# Information maintenance in working memory: an integrated presentation of cognitive and neural concepts

**DOI:** 10.3389/fnsys.2015.00104

**Published:** 2015-07-14

**Authors:** Markus Martini, Marco R. Furtner, Thomas Maran, Pierre Sachse

**Affiliations:** Department of Psychology, Leopold-Franzens University of InnsbruckInnsbruck, Austria

**Keywords:** working memory, information maintenance, states of activities, phase synchronization, frequency bands, molecular biological mechanisms

## Abstract

Working memory (WM) maintains information in a state that it is available for processing. A host of various concepts exist which define this core function at different levels of abstraction. The present article intended to bring together existing cognitive and neural explanatory approaches about the architecture and neural mechanisms of information maintenance in WM. For this, we highlight how existing WM concepts define information retention and present different methodological approaches which led to the assumption that information can exist in various components and states. This view is broadened by neural concepts focussing on various forms of phase synchronization and molecular biological mechanisms relevant for retaining information in an active state. An integrated presentation of different concepts and methodological approaches can deepen our understanding of this central WM function.

Cognitive abilities such as speaking, writing, navigating, reasoning, planning, and problem solving can all be related to a cognitive construct called working memory (WM). WM maintains information and makes it available for processing (Oberauer and Hein, 2012). We intended to look at this core function from central cognitive and neural angles. Specifically, the present article brings together various explanations about what is understood by information retention. Therefore, we focus on various WM concepts and how the maintenance function is implemented and investigated. This is followed by highlighting neural concepts which base their assumptions on findings that short-term maintenance can be viewed as synchronized neural activity and present relevant molecular biological mechanisms which were identified when information is retained in an active state.

## Cognitive Concepts of Information Maintenance

How can WM be defined and what role plays the maintenance function within it? For Baddeley (2003, p. 829) “the concept of WM proposes that a dedicated system maintains and stores information in the short term, and that this system underlies human thought processes”. Jonides et al. (2005, p. 2) define it as “system that can store a small amount of information briefly, keeping that information quickly accessible and available for transformation by rules and strategies, while updating it frequently”. Postle (2006, p. 23) refers WM “… to the retention of information in conscious awareness when this information is not present in the environment, to its manipulation, and to its use in guiding behavior”. Jarrold and Towse (2006, p. 39) define WM “… as the ability to hold in mind information in the face of potentially interfering distraction in order to guide behavior. Finally, Burgess et al. (2011, pp. 674–675) refer WM to “… a limited cognitive system involved in the temporary storage and manipulation of information required for task-relevant performance … In particular, the cognitive construct of WM can likely be decomposed into domain-specific mechanisms that govern the active maintenance of information over short periods of time, and domain-general central executive processes that regulate and coordinate those maintenance operations”. The various definitions of WM make clear that this construct incorporates several functions but one of the most prevalent is that a limited amount of information is temporally maintained in an active state.

**Figure 1:**
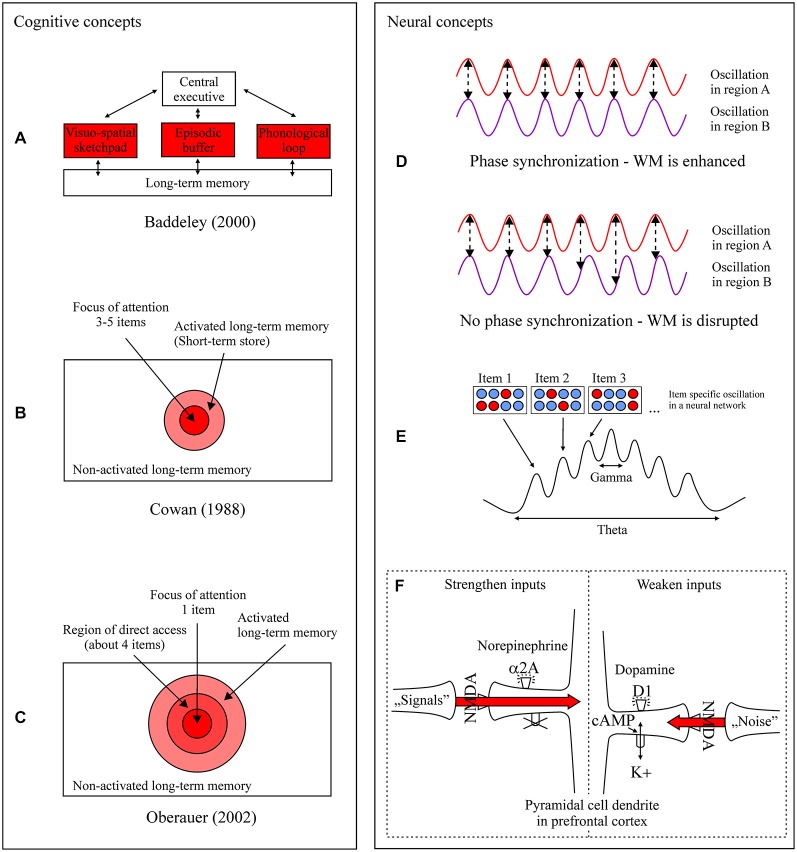
**Different cognitive (A–C) and neural (D–F) concepts of short-term information maintenance. (A–C)** Components and states in which information can be actively maintained are highlighted in red. For details to the three models, consult the text. **(D)** Phase synchronization within and between specific cell ensembles and brain regions (e.g., frontal cortex and parietal cortex regions) heightens the probability of correct encoding, maintenance, and retrieval of information compared to no phase synchronization (from Fell and Axmacher, 2011). In synchronized oscillatory activity the oscillatory activity in region A peaks of oscillatory activity in region B, resulting in an efficient information transfer and higher working memory (WM) performance. This pattern is not found in non-synchronized oscillations (no phase synchronization), resulting in an inefficient information transfer and lower WM performance. **(E)** WM processes are related to oscillatory processes in the theta and gamma frequency range. Here, within the same network (blue and red circles) different items are active within different groups of cells (red). Items held in WM are represented in different gamma cycles nested within different phases of a theta cycle. The information is maintained in WM through the repetition of the specific theta-gamma frequency pattern (from Lisman, 2010). **(F)** Shown is a simplified representation of information maintenance at the molecular level in prefrontal cortex circuits. The catecholamines norepinephrine and dopamine have high influences on the functional strength of network synapses. Norepinephrine activation of α2A receptors on spines inhibits subsequent cyclic adenosine monophosphate (cAMP) production. Nearby potassium channels are closed and as a result the strength of a signal is increased. On the other hand, on a different set of spines a noise signal can be switched off. A noise signal is controlled for through the production of cAMP through dopamine and D1 receptor activity. As a consequence of cAMP activity nearby potassium channels are opened. For maintaining a clear information signal, optimal levels of transmitter release and cAMP production are of central relevance. Excessive cAMP production (e.g., stress) disconnects all network inputs and shuts down cell firing resulting in information loss (from Arnsten and Rubia, 2012).

A host of dominating WM concepts exist which depict the maintenance function at different levels of explanation and methodological approach. For Baddeley and Hitch (1974) and later Baddeley (2000) information is held in three stores which strongly interact with long-term memory (LTM) and an attentional system, the central executive (Figure 1A). Depending on the content, information is maintained in capacity limited short-term buffers. Maintenance of phonological information (e.g., words) is organized within the phonological loop, consisting of a passive phonological store and an active subvocal rehearsal process, which refreshes information in this store (e.g., Baddeley, 2003). Visual (e.g., color) and spatial (e.g., location) information is maintained in the visuo-spatial sketchpad (e.g., Klauer and Zhao, 2004). It was proposed that the maintenance of visuo-spatial information is organized within a passive visual store (called the visual cache) and an active process (called the inner scribe) responsible for processing, manipulating, and refreshing visuo-spatial information including movements (Logie, 1995). However, the precise nature of visuo-spatial rehearsal remains unclear (Baddeley, 2012). The episodic buffer represents a buffer store for all the components of WM. Specifically, it holds a limited number of integrated episodes or chunks (about four chunks) in a multidimensional code (e.g., when reading a story, pictures or scenes are generated at the same time). Whether a specific refreshing mechanism exists and how it works is unclear (Baddeley, 2012). The basic and primarily applied approach to identify the various components and its subcomponents is the dual task paradigm. The principle of this paradigm is that two tasks have to be processed at the same time. For instance, holding in mind a certain amount of information (e.g., numbers, letters, patterns) while at the same time a different task is processed (e.g., calculations, logical reasoning, or categorization tasks). With this methodological approach one can investigate whether a process works or not when another one is heavily loaded, systematically decomposing the components of WM.

Different structural concepts of WM propose varying states of activity that information can take on when it is maintained for a short period of time. The fundamental basis of these hierarchically organized models is LTM, based on which information can be actively maintained in different states in WM. Cowan (1988, 1995, 1999, 2005; Figure 1B) assumes that information can take on three states in mind, non-active, temporally active within LTM, and information within the focus of attention (FA) as a subset of active memory. Information maintained in the FA has the highest state of activity. Only a limited amount of information, between three to five items, is retained in the FA in a relatively stable state (Cowan, 2001). Information outside the FA and within the activated LTM is less stable and decays within seconds when it is not actively refreshed through active rehearsal or attentional refreshing mechanisms. The extension of two activity states by a third one was proposed by Oberauer (2002, 2009; Figure 1C). For Oberauer information can be maintained in: (i) the component of the activated part of LTM (aLTM); (ii) the component for maintaining new structural representations (about four items or chunks) by dynamic relational bindings, called the direct access region (DAR; also termed the broad FA); and (iii) the FA, which selects one single item or chunk (also termed the narrow FA; Oberauer and Hein, 2012).

Various findings based on different paradigms led to the assumption that maintenance can be distinguished between different states of activity. Two applied paradigms are the change detection task (e.g., Luck and Vogel, 1997) and the two-cue, two-probe (2C2P) recognition task (e.g., Oberauer, 2005). Various versions of the change detection task exist. In one version information elements (e.g., five squares in different colors) are simultaneously presented for a short time. After a delay period a probe display is presented and participants have to indicate whether something has changed or not. Based on this kind of task it was proposed that within the FA about three to five items or chunks can be actively maintained. However, through a simple manipulation based on a retro cue, findings indicate that the maintenance capacity of the FA is much more limited. A retro cue marks a possible change in the upcoming probe display and decreases participants’ probe reaction times. Facilitation is only found with one retro cue and not with two retro cues (Makovski and Jiang, 2007) further supporting the one-item FA view. With the 2C2P recognition task (Oberauer et al., 2003; Oberauer, 2005) distinguished the maintenance states of the aLTM and the DAR. The principle of the task is that participants have to memorize differently marked information (e.g., two word lists, A and B, in the colors blue and red). The information display is followed by a cue (e.g., red frame), which indicates what information becomes relevant, before a recognition decision, the probe (e.g., one word of two lists, or a new one), has to be made. This decision is followed by a second cue and a second probe for the same information display in this trial. The FA allocation can be experimentally manipulated through variation of the: (i) amount of information which has to be maintained (e.g., one vs. three words per list); (ii) cues (e.g., list A or B is relevant); (iii) cue-probe interval (e.g., few milliseconds vs. seconds); and (iv) probe content (probe-lists matches or new word). The different activity states are identified by varying reaction times to the probe. For instance, probe reaction times increase when the first cue and the first probe do not match or the second cue indicates that a prior as irrelevant cued list (by the first cue) becomes now relevant (Oberauer, 2005; Oberauer and Hein, 2012).

In sum, dominating WM models describe active maintenance at various levels and states. The maintenance of information within WM is often described as activity, attentional, or subvocal rehearsal. However, how the various maintenance mechanisms function and how they work on a neural level is unclear (Baddeley, 2012).

## Neural Concepts of Information Maintenance

### Electrophysiological Correlates of Information Maintenance and Neural Oscillation

Neurophysiological data shed additional light on the maintenance function and give further support in understanding how our brain retains information. Information that is held over a short period of time in WM is represented in an activated neural network of interconnected nerve cells in prefrontal and various posterior cortical and subcortical regions (e.g., Öztekin et al., 2009; Nee and Brown, 2013). For instance, neuroimaging studies found that WM tasks led to an increase in activation of ventrolateral, dorsolateral, medial, and parietal cortex areas and this increase was higher for high span individuals compared to low span individuals (e.g., Osaka et al., 2004, 2007; for a review see D’Esposito and Postle, 2015). Additionally, different states of maintenance have been found based on pattern classifier methods of blood-oxygen-level-dependent (BOLD) signals. Lewis-Peacock et al. (2011) found that only the content of a relevant cue was reflected in ongoing neuronal activity. Irrelevant content dropped to baseline, but the specific pattern reappeared when it became again relevant. This finding speaks for at least two maintenance states, neuronal active information within the FA, and information represented within decreasing activity outside the FA, but in a state in which the represented information and its relational bindings can be reactivated (McElree, 2001).

During maintenance nerve cells communicate and various molecular mechanisms are active. One assumption about how our brain retains information over short periods of time is through phase synchronization of active nerve cells. This means that specific cell ensembles within and between different brain regions start to fire in a coordinated, oscillatory manner, repeatedly or for an extended period of time (Figure 1D). Studies in humans and animals can show that phase synchronization is positively related with memory recall and is discussed as a mechanism to induce synaptic plasticity, a cellular mechanism for strengthening the connectivity between nerve cells to generate longer lasting memories called long-term potentiation (Huerta and Lisman, 1993; Fell and Axmacher, 2011). Results indicate that the maintenance of information is related to theta band (4–8 Hz; e.g., Jensen and Tesche, 2002), alpha band (8–12 Hz; e.g., Jensen et al., 2002), beta band (13–30 Hz; e.g., Axmacher et al., 2008), and gamma band synchronization (30–120 Hz; e.g., Howard et al., 2003). Various findings of neuronal oscillation studies can show that specific frequency bands and cross frequency couplings can be related to different WM functions relevant for maintaining information (Freunberger et al., 2011). Gamma oscillations can be related to maintenance of WM information and attentive processing of information (Fries et al., 2001; Roux and Uhlhaas, 2014). Inhibition of irrelevant information, gating of sensory information, and prevention of interference from distracting stimuli can be related to alpha oscillations (e.g., Zumer et al., 2014; Herrmann et al., 2015). The selection of maintained information (e.g., task rules) and long range synchronization (e.g., between prefrontal and posterior parietal cortex areas) are related to beta oscillations (Buschman and Miller, 2007; Buschman et al., 2012; Dipoppa and Gutkin, 2013). Specifically, long-range synchronization of different frequencies have been asscociated with top-down control, WM content manipulation, and synaptic plasticity (Schack et al., 2005; Fell and Axmacher, 2011; Crespo-Garcia et al., 2013), and seem to be synchronized by different sub-regions of the thalamus (Ketz et al., 2015). When items are held active in the correct order, during a delay period before recall, results indicate that theta and gamma frequency bands are coupled (i.e., phase locked). In this state single items are presented in gamma cycles nested within a theta cycle (Figure 1E; e.g., Jensen and Lisman, 2005; Lisman, 2010). Varying amounts of maintained information is organized through theta frequency band shifts in that an increase in WM load leads to a shift to lower theta frequencies and* vice versa* (e.g., Jensen and Tesche, 2002; Axmacher et al., 2010). Specifically, medial frontal theta was shown to be related to the temporal order maintenance of items compared to items maintenance alone (Hsieh et al., 2011; Roberts et al., 2013). In contrast, alpha frequencies tend to occur in tasks that require the retention of simultaneously presented information (Roux and Uhlhaas, 2014). In reviewing the literature Roux and Uhlhaas (2014) presented a framework of oscillatory networks for sequential and sensory WM. Sensory information is presented via alpha-gamma oscillations in predominantly areas like the prefrontal cortex, parietal cortex, and the thalamus. Whereby gamma oscillations underlie active maintenance and read-out of relevant WM items, and alpha oscillations underlie the inhibition of task-irrelevant WM information. Sequential information is presented via theta-gamma oscillations in predominantly areas like the medial temporal lobe and frontal cortices.

The various findings show that for maintaining information in WM various cell ensembles and brain regions synchronize. Nee and Jonides (2013a), supporting the three-state view of Oberauer (2002), found that access of (visual) information held in the FA activated the ventral posterior parietal cortex, access of items in the DAR activated the medial temporal lobe, and access to items in the aLTM activated the ventrolateral prefrontal cortex. In their neural three-state model, Nee and Jonides (2013b) specified their view proposing that frontal and posterior areas interact to represent information in short-term memory. The posterior region of intraparietal sulcus maintains spatial information and is strongly connected to the frontal region of superior frontal sulcus. Object information is maintained in the posterior region of inferior temporal cortex and is connected with the frontal region of the ventrolateral prefrontal cortex. The medial temporal lobe is responsible for embedding the items into a context and is connected to both frontal areas. Nee and Jonides (2013b) assume that phase synchronization (theta-gamma couplings) between these frontal and posterior areas form the basis for the maintenance of item and location information, while synchronization of the medial temporal lobe and posterior areas form the basis for the maintenance of item-location bindings.

Future studies have to further confirm the view that WM represents an activated part of LTM and clearly define the differences to a WM buffer view. Evidence exist that specific parts of the hippocampus (e.g., CA1 region) and the surrounding medial temporal cortices (e.g., entorhinal and perihinal cortex) can maintain information in co-exitence of LTM processes in a distractor resistant state (e.g., Suzuki et al., 1997; Schon et al., 2004, 2015; Jensen and Lisman, 2005). In addition, future studies have to investigate spatiotemporal oscillatory dynamics of encoding, maintenance, and retrieval phases. Recent research indicates that these phases represent functionally discrete and measurable subprocesses (e.g., electrophysiological correlates of encoding and maintenance, Heinrichs-Graham and Wilson, 2015; refreshing, Johnson et al., 2015).

### Molecular Biological Correlates of Information Maintenance

Beside electrophysiological and neuroimaging data, there is strong progress in describing the molecular mechanisms of short-term maintenance. Neural models (i.e., the simulation of neurobiological processes in an artificial neural network), pharmacological manipulations (e.g., blocking a receptor and therefore disrupting memory performance, by an antagonist which imitates a specific transmitter), and genetic manipulations (e.g., knockout (KO) mice, which do not express a particular protein, leading to a disruption of memory formation, and technics like optogenetics, or designer receptors and transmitters) strongly increased our understanding how our memory operates at the molecular level (e.g., Reijmers and Mayford, 2009; Rolls et al., 2013; Kandel et al., 2014).

Within the prefrontal cortex the neurotransmitters and respective receptors of glutamate, norepinephrine, and dopamine play an important role in generating a neural signal representing information maintained in WM (e.g., a picture or letter that is no longer externally present; e.g., Clark and Noudoost, 2014). Specifically, in the dorsolateral prefrontal cortex of primates persistent activity is generated through recurrent excitation microcircuits through glutamate in pyramidal delay cells, which maintain persistent firing in the delay period of a WM task (Figure 1F). Studies found that blocking of the glutamate N-methyl-D-aspartate (NMDA) receptors but not α-amino-3-hydroxy-5-methyl-4-isoxazolepropionic acid (AMPA) receptors, can abolish persistent firing and consequently diminishes WM performance (Wang et al., 2013).

In addition to glutamate, dopamine and norepinephrine affect WM functioning. Evidence exists that dopamine plays a specific role in maintaining information in WM within the prefrontal cortex and gating (updating) the maintained information via the striatum (Cools and D’Esposito, 2011). Transmitter release follows an inverted U-shaped performance curve, i.e., too high or low transmitter levels impair WM performance (Arnsten, 2011; D’Ardenne et al., 2012). Whereby a neurochemical reciprocity between prefrontal cortex and striatum exists. This means that increases and decreases in prefrontal dopamine decreases and increases striatal dopamine levels (Cools and D’Esposito, 2011). Transmitter release influences thereby the functional strength of a synapse. Studies found that through the noradrenergic adrenergic (α2A) receptors the production of an intracellular messenger called cyclic adenosine monophosphate (cAMP) that can open and close ion channels, is inhibited. This cAMP inhibition leads to a closing of nearby potassium channels resulting in an increase in the connection strength of the network. On the other hand, the activation of dopaminergic (D1) receptors leads to the opening of potassium channels through cAMP, resulting in a removal of an action potential. Taken together, to hold a signal in a stable state the activation of NMDA channels together with α2A-cAMP interactions to forward correct signal inputs, and D1-cAMP interactions to inhibit noise signals seem to be of relevance when information is maintained in specific prefrontal cortex regions (e.g., deep layer III; Arnsten et al., 2012). However, holding information active via spikes is energetically expensive because an action potential has high metabolic costs. Therefore, a much more effective way of holding information in an active state is to increase the residual calcium levels at presynaptic terminals (e.g., Kandel and Segelbaum, 2000; Mongillo et al., 2008). Accumulation of calcium takes place because removal of residual calcium from presynaptic terminals is a relatively slow process. Therefore, more calcium is present to cause presynaptic transmitter release without the need for action potentials. Mongillo et al. (2008) indicate that through this process information can be transiently held for about 1 s without enhanced spiking activity. This view is in line with recent neural models that can show that by the mechanisms of synaptic facilitation the number of short-term memories that can be retained in WM can be increased. In this context Rolls et al. (2013) found that a key parameter was the inhibitory synaptic weights, which play a specific role in “tuning” a neural signal, resulting in a clearer WM representation (but see Wang et al., 2015, who found an inhibitory persistent activity interneuron). Rolls et al. (2013) argue that the number of short term memory representations that can be simultaneously maintained in active state depends on the tuning of the inhibition parameter. They found that this tuning becomes increasingly important when capacity limits are reached, i.e., when seven or more memories have to be retained. Therefore, an optimal excitation/inhibition balance is important. For stabilizing information in WM a balanced microcircuity of excitation and inhibition is needed. In this context, positive and negative feedback loops can stabilize the WM content. Regarding this, excitatory to excitatory connections represent a positive feedback, and excitatory to inhibitory connections drive a negative feedback. Lim and Goldman (2013) found that the former connection produces a slow and the latter a fast signal leading to a corrective, negative-derivative form of feedback that counteracts drift in persistent activity which leads to a more stable signal.

Finally, holding information in mind can heighten the probability that this information is transferred to LTM. In this context, long-term potentiation describes a mechanism in which nerve cells build new synaptic connections to transfer short-term information into longer lasting memories. For instance, it was found that a high frequency excitation of hippocampus neurons in rats can increase synaptic strength for hours (e.g., Bliss and Lomo, 1973). In early phases of long-term potentiation additional AMPA receptors are inserted in the postsynaptic cell membrane as response to heightened presynaptic transmitter release based on strong (tetanic) excitation (Squire and Kandel, 2009). Specifically, the Glutamate A1 (GluA1) subunit of the AMPA receptor seem to be necessary for short-term memory (or at least the expression of short-term memory) but not for long term memory (Sanderson and Bannerman, 2011; Sanderson et al., 2011). Another mechanism was found in the Schaffer collaterals of the hippocampus. Findings indicate that a tetanic activity of 200 Hz induces a long-term potentiation by which the presynaptical release of neurotransmitters is facilitated through a retrograde messenger. In other words, a strong excitation of the postsynapse caused a gas, nitric oxide (NO), to wander from the postsynapse to the presynapse to facilitate transmitter release. Further, this mechanism was only then effective when there was a coincidence of NO and presynapse activity. These results were not found under a tetanic activity of only 100 Hz (Wang et al., 2005). Thus, the postsynapse adapts to a strong signal and maintains it through different mechanisms (Sakurai, 1990; for a role of short-term potentiation in short-term memory, e.g., Erickson et al., 2010).

## Conclusion

WM is needed whenever we try to hold information in mind, in a highly accessible state (e.g., a thought, scene, or just a name), partly in parallel of different other ongoing processes (e.g., speaking, listening, planning, and problem solving; Miller and Desimone, 1994; Martini et al., 2015). The outlines above show that the concept of information maintenance in WM is complex and that it can be defined differently at multiple levels of explanation. On a cognitive level views exist that information can be maintained inside the FA and outside the FA, whereby capacity limitations, accessibility, and stability vary between the different states. Other views prefer a characterization of information maintenance in specialized components, describing specific mechanisms of retention. Neuroimaging and electrophysiological studies have further enlightened where and how information is maintained in our brain. These studies can show that information is maintained in a distributed network of functionally specialized brain areas (e.g., frontal, parietal, and medial temporal lobe; Nee and Jonides, 2013b). Within and between these brain areas information is maintained in a network of active nerve cells which communicate in a language of oscillatory patterns. This means, information is retained in different frequency bands which can be coupled (e.g., theta-gamma coupling; Lisman, 2010) to maintain a limited amount of information in a way that every retained information element is precisely coded. At the lowest level of explanation information maintenance is described by molecular biological activity. Depending on the brain area partly different molecular biological mechanisms are responsible for retaining information in the neural network (e.g., maintaining mechanisms in the lateral prefrontal cortex differ from mechanisms in the hippocampus; Arnsten et al., 2012). For a clear neural signal to be generated the neural network must be tuned in way that the correct input remains active and noise is controlled for, and that an optimal balance of excitatory and inhibitory activity is ensured. Future research will help to gain deeper insights regarding the interplay of relevant cognitive concepts and basic neural mechanisms responsible for the fundamental cognitive function of information maintenance in WM. This issue has to be discussed in the light of the information type (declarative vs. procedural, e.g., Oberauer, 2009, 2010), how resources are allocated between retained items (e.g., every item gets the same vs. different amounts of resources; Ma et al., 2014), and the information flow between different stores (e.g., from perceptual to prefrontal regions; Sreenivasan et al., 2014).

## Author Contributions

MM, MRF, TM, and PS wrote the manuscript. All authors reviewed the manuscript.

## Conflict of Interest Statement

The authors declare that the research was conducted in the absence of any commercial or financial relationships that could be construed as a potential conflict of interest.
